# A Secreted Chorismate Mutase from *Xanthomonas arboricola* pv. *juglandis* Attenuates Virulence and Walnut Blight Symptoms

**DOI:** 10.3390/ijms221910374

**Published:** 2021-09-26

**Authors:** Renata de A. B. Assis, Cíntia H. D. Sagawa, Paulo A. Zaini, Houston J. Saxe, Phillip A. Wilmarth, Brett S. Phinney, Michelle Salemi, Leandro M. Moreira, Abhaya M. Dandekar

**Affiliations:** 1 Department of Plant Sciences, University of California, Davis, CA 95616, USA; redab@ucdavis.edu (R.d.A.B.A.); chdsagawa@ucdavis.edu (C.H.D.S.); pazaini@ucdavis.edu (P.A.Z.); hsaxe@ucdavis.edu (H.J.S.); 2 Departamento de Ciências Biológicas, Instituto de Ciências Exatas e Biológicas, Núcleo de Pesquisas em Ciências Biológicas, Universidade Federal de Ouro Preto, Ouro Preto 35400-000, MG, Brazil; 3 Proteomics Shared Resource, Oregon Health and Science University, Portland, OR 97239, USA; wilmarth@ohsu.edu; 4 Proteomics Core Facility, University of California, Davis, CA 95616, USA; bsphinney@ucdavis.edu (B.S.P.); msalemi@ucdavis.edu (M.S.)

**Keywords:** *Xanthomonas*, *Juglans regia*, walnut blight, secreted chorismate mutase, TMT-plex, proteome, hypervirulence

## Abstract

Walnut blight is a significant above-ground disease of walnuts caused by *Xanthomonas arboricola* pv. *juglandis* (Xaj). The secreted form of chorismate mutase (CM), a key enzyme of the shikimate pathway regulating plant immunity, is highly conserved between plant-associated beta and gamma proteobacteria including phytopathogens belonging to the Xanthomonadaceae family. To define its role in walnut blight disease, a dysfunctional mutant of chorismate mutase was created in a copper resistant strain Xaj417 (XajCM). Infections of immature walnut *Juglans regia* (Jr) fruit with XajCM were hypervirulent compared with infections with the wildtype Xaj417 strain. The in vitro growth rate, size and cellular morphology were similar between the wild-type and XajCM mutant strains, however the quantification of bacterial cells by dPCR within walnut hull tissues showed a 27% increase in XajCM seven days post-infection. To define the mechanism of hypervirulence, proteome analysis was conducted to compare walnut hull tissues inoculated with the wild type to those inoculated with the XajCM mutant strain. Proteome analysis revealed 3296 Jr proteins (five decreased and ten increased with FDR ≤ 0.05) and 676 Xaj417 proteins (235 increased in XajCM with FDR ≤ 0.05). Interestingly, the most abundant protein in Xaj was a polygalacturonase, while in Jr it was a polygalacturonase inhibitor. These results suggest that this secreted chorismate mutase may be an important virulence suppressor gene that regulates Xaj417 virulence response, allowing for improved bacterial survival in the plant tissues.

## 1. Introduction

**Figure 1:**
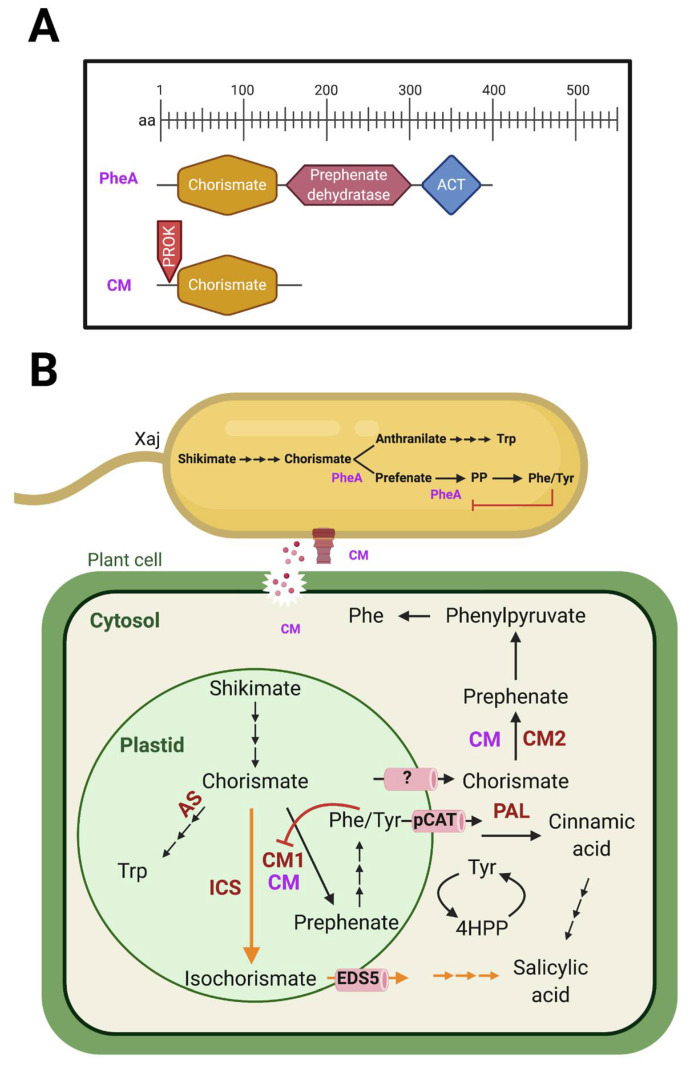
(**A**) Bacterial PheA and CM domain composition. (**B**) Shikimate pathway in phytopathogens and plants. Red: plant enzymes. Purple: bacterial enzymes. PheA: prephenate dehydratase; Trp: tryptophan; PP: phenylpyruvate; Phe: phenylalanine; Tyr: tyrosine; CMs: secreted chorismate mutase; AS: anthranilate synthase; ICS: isochorismate synthase; CM1: plastidic chorismate mutase; CM2: cytosolic chorismate mutase; pCAT: plastidial cationic amino acid transporter [7]; EDS5: enhanced disease susceptibility 5 [8]; PAL: phenylalanine ammonia-lyase; 4HPP: 4-phenylpyruvate. In this model the bacterial secreted CM augments prephenate synthesis, while consuming precursors of salicylic acid, thus dampening plant immunity. Created in BioRender.com with information from Djamei et al. 2011, Qian et al. 2019, and Lefevere et al. 2020 [5,7,8].

Chorismate is the common intermediate for the production of primary and secondary compounds such as aromatic amino acids, vitamins, and phytohormones such as salicylic acid (SA). Chorismate mutase (CM) is responsible for the conversion of chorismate to prephenate (Figure 1). In plants, salicylic acid (SA) stimulates the systemic plant immune response to pathogens, and it is produced from the precursor chorismate via isochorismate synthase. In bacteria, SA is an intermediate in the biosynthesis of siderophores to scavenge iron [1]. Interestingly, the shikimate pathway is present in bacteria, fungi, algae, and plants but not in animals [2]. Plants and microorganisms share enzymes from the shikimate pathway responsible for the synthesis of vitamins, aromatic amino acids and other secondary metabolites [2]. In addition, bacteria have an enzyme responsible for the conversion of chorismate to 4-hydroxybenzoate and can produce SA either by a bifunctional SA synthase enzyme or by two separate enzymes: isochorismate synthase (conserved in plants) and isochorismate pyruvate lyase (only found in bacteria) [1].

The chorismate mutase enzyme catalyzes a key reaction in the shikimate pathway involved in the biogenesis of amino acids such as tyrosine and phenylalanine (Figure 1). Plants have at least two chorismate mutase enzymes present in different cellular locations. Allosteric regulation is a characteristic feature of plastidic chorismate mutases, whereas cytosolic plant chorismate mutases lack this feature [3]. Under stress, conversion to isochorismate is favored to produce SA. Phytopathogens from the Xanthomonadaceae family have two enzymes that share a chorismate mutase domain (Figure 1A). These enzymes belong to two different protein families [4]. PheA (chorismate mutase/prephenate dehydratase) is responsible for aromatic amino acid synthesis for bacteria metabolism, while CM is secreted [4]. The introduction of CM into plants may possibly interfere with this pathway and limit the availability of chorismate that can be converted to salicylic acid, altering the plant defense response during the early phases of infection. In *Ustilago maydis*, Cmu1 is homologous to CM and spreads to neighboring cells redirecting the host metabolome through metabolic priming [5]. The increased flow of chorismate from plastid to the cytosol lowers available substrate for SA biosynthesis in plastids. The removal of CM in the causal agent of rice leaf blight *Xanthomonas oryzae* pv. *oryzae* resulted in bacterial hypervirulence in rice [6]. These studies suggested the crucial role of CM in controlling bacterial virulence, however, the mechanisms involved in the plant response are still unknown.

Xaj is the causal agent of walnut blight (WB), a relevant disease that affects walnut production worldwide. In California, the overuse of copper pesticides to control the spread of the disease has led to the emergence of copper-resistant strains such as Xaj417 [9,10]. A detailed comparative genomics study using Xaj417 as a reference demonstrated that this strain acquired a new copper resistance cassette by lateral gene transfer, associated with a new transposon family in *Xanthomonas* [10]. This study also showed that an expansion of mobile genetic elements (MGE) among these pathogenic strains also influenced the repertoire of virulence factors and thus adaptation strategies. MGE enrichment in Xaj pathogenic strains may be correlated with the horizontal dissemination of virulence factors shaping the genome structure of different strains. The absence of Transcription Activator-like Effectors (TALEs) in Xaj strains suggests a distinct mechanism of virulence induction and modulation of plant responses [11]. We dissected this pathosystem using tandem mass tag quantitative proteomics, demonstrating the expression of CM and other type II secretion effectors during WB [12], and now we will expand our analysis to understand the specific role of CM during infection. We hypothesize that CM modulates the plant immune system by decreasing SA production while increasing aromatic amino acid production and iron sequestration via the production of siderophores. Our aim was to assess the effect of a dysfunctional mutation in the conserved secreted CM protein in the copper-resistant strain Xaj417 of the plant-associated Xanthomonadaceae [4].

## 2. Results

Xaj417 has two different genes with distinct domain composition and regulation that share a chorismate mutase domain (Figure 1) [4]. PheA has three domains responsible for the conversion of chorismate to phenylalanine and tyrosine that is allosterically regulated by the concentration of these amino acids. CM is non-allosteric, has a signal peptide, and a domain responsible for the conversion of chorismate to prephenate. We generated a dysfunctional mutation in XajCM by inserting a functional kanamycin resistance gene into the active site region of the gene encoding this enzyme. We confirmed the bacterial mutation by growth in antibiotic selection media, PCR and DNA sequencing (Figure S1).

### 2.1. Analysis of the Mutation Effect of XajCM on Bacterial Growth and Cell Morphology

**Figure 2:**
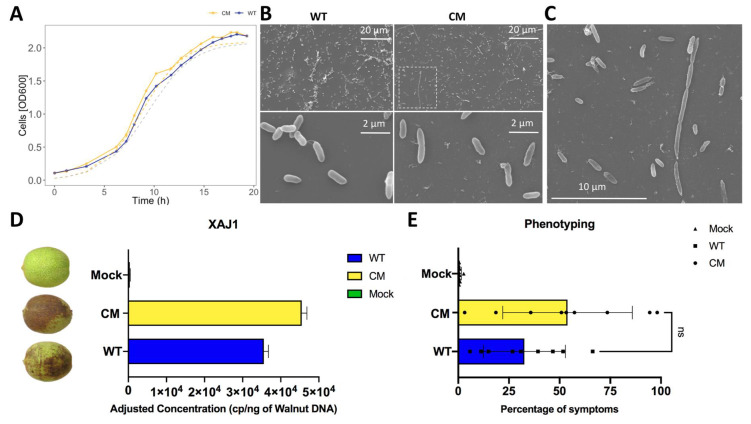
(**A**) Xaj WT and XajCM mutant growth curves. Dotted lines represent predicted OD values according to the model of growth for the samples analyzed. (**B**) In vitro microscopy of Xaj WT (left) and XajCM (right) cultures, magnification of 1200× (top) and 12,000× (bottom). (**C**) XajCM linear shape forming a chain of individual cells similar to a streptobacilli form (magnification of 3500×). (**D**) Quantification of Xaj in plant tissue by dPCR and (**E**) Walnut blight phenotyping in walnut fruits using ImageJ.

The quantification of the bacterial growth in vitro showed that the mutation displayed a normal growth rate in rich media (Figure 2A). However, we observed changes in the bacteria morphology between XajCM mutant and WT strains. The XajCM mutant showed similar size and shape compared to Xaj WT but showed a peculiar linear organization forming a chain of individual cells similar to streptobacilli (Figure 2B,C).

Walnut hulls were inoculated with XajCM mutant and Xaj WT, incubated at room temperature with controlled light, and plant response was evaluated for 7 to 10 days. Although inoculated with the same initial concentration of cells, we observed different growth rates in planta by quantifying the total number of bacterial cells in infected walnut tissue 7 days after inoculation. XajCM showed an increase of 27% compared to WT (Figure 2D,E). Inoculations of walnut fruits with XajCM showed a greater percentage of symptoms in hull tissues (Figure 2D). The infected walnut fruit were incubated in a 12 h light/dark cycle to simulate field conditions. The side of the walnut fruit facing the light showed fewer symptoms in all treatments as compared to the side facing away (Figure S2).

### 2.2. Proteomic Analysis of XajCM in the Walnut Hull

To identify host proteins associated with blight disease susceptibility, we conducted a proteomic analysis of walnut hull tissues inoculated with Xaj WT and the mutant strain XajCM, respectively. The proteins were detected based upon their abundance in the tissue extract and the TMT labeling enabled the estimation of the relative abundance of individual proteins during infection and disease development [13,14]. Pooled samples of hull tissue inoculated with XajCM, Xaj WT, and mock were collected, and total protein concentrations were adjusted to 1 μg/μL. A total of 3972 (3296 Jr and 676 Xaj417) proteins at a minimum of two peptides at a 5% False Discovery Rate (FDR) and a maximum of ten decoys were detected (Tables S1 and S2).

**Figure 3:**
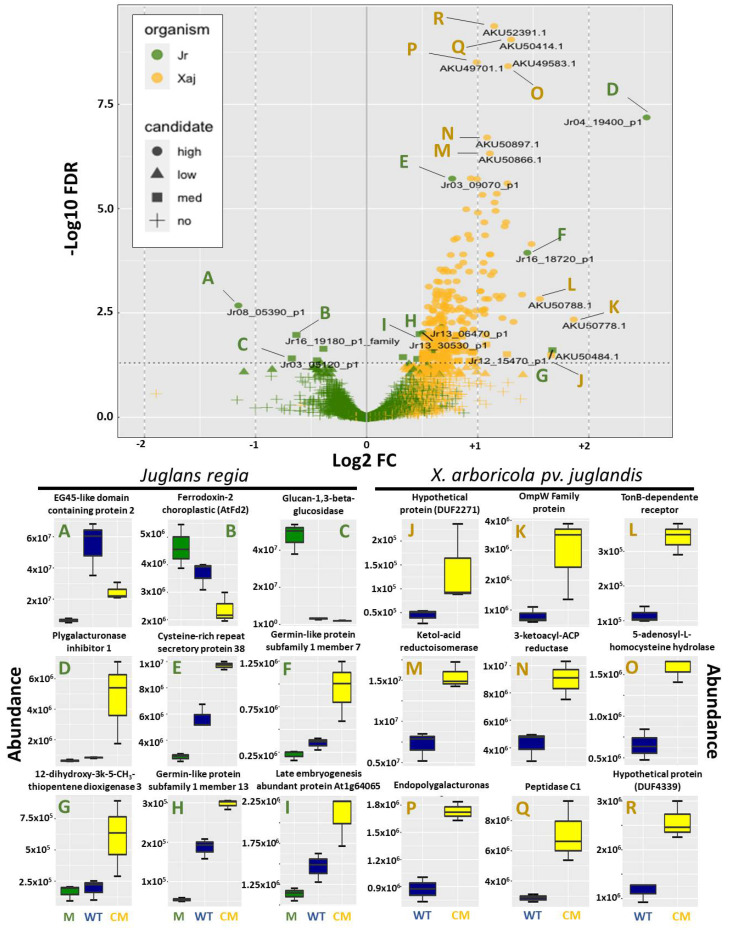
Volcano plot highlighting differentially abundant proteins with FDR ≤ 0.05 and protein abundance comparisons among inoculations of hull tissue with Mock (**M**), Xaj WT (WT) and XajCM (CM) of Jr (**A**–**I**) and Xaj (**J**–**R**) proteins. (**L**) TBDR AKJ12_14110.

Partial least squares-discriminant analysis (PLS-DA) evaluates the Variable Importance in Projection (VIP) score to estimate the importance of each variable in the projection used in a PLS model, and is often used for variable selection. Important proteins responsible for group discrimination in Xaj417 and *J. regia* in response to infection were identified by PLS-DA (Figures S3–S6). Among the 676 Xaj proteins identified, 251 with VIP higher than 1 were increased in the XajCM in comparison with Xaj WT. In addition, 826 *J. regia* proteins with VIP higher than 1 were identified among the total of 3296 Jr proteins. Compared with Xaj WT, 668 proteins were increased in XajCM (235 with FDR ≤ 0.05, and 433 with FDR > 0.05)—Figure 3 and Table S3. Only eight Xaj proteins were decreased (FDR > 0.05), including the secreted chorismate mutase, further confirming that the expression of this enzyme is indeed knocked out in the mutant strain (Figure S1C). The differentially abundant proteins with FDR ≤ 0.05 in Jr and Xaj were highlighted in the volcano plot (Figure 3A-R). Protein abundance levels were compared between plants inoculated with the WT and XajCM mutant. The volcano plot analysis of *J. regia* proteins showed that the EG45-like domain-containing protein was the most decreased protein during infection of XajCM (Figure 3A) together with glucan-1,3-beta-glucosidase and ferredoxin (Figure 3B–C). Prediction of protein–protein interactions using STRING showed that EG45-like protein from *Arabidopsis* (AT2G18660.1) may interact with glucan-1,3-beta-glucosidase [15]. The inhibitor-like protein polygalacturonase was identified as the most abundant protein in *J. regia* (Figure 3D) and endo-polygalacturonase as the most abundant in Xaj (Figure 3P) during infection. The other proteins that increased in Jr with FDR ≤ 0.05 were a cysteine rich-repeat secretory protein 38, germin-like protein subfamily 1 members 7 and 13, 1,2-dihydroxy-3-keto-5-methylthiopentene dioxygenase 3, and a late embryogenesis abundant protein (Figure 3E–I). The 35 top proteins identified by PLS-DA in Xaj417 include OmpW (Figure 3K), polygalacturonase, XopX, isochorismatase, five TonB-dependent receptors—TBDR (AKJ12_RS14110/RS14490/RS14670/RS06190/RS07080), and five hypothetical proteins (AKJ12_RS14105/RS21575/RS07070/RS18130/RS01945). Finally, other proteins increased among Xaj proteins with FDR ≤ 0.05 were a keto-acid reductoisomerase, 3-ketoacyl-ACP reductase, 5-adenosyl-l-homocysteine hydrolase, and a peptidase C1 (Figure 3M–R).

Among the hypothetical proteins, 14 have FDR ≤ 0.05 and were increased in the mutant (Table 1). AKJ12_14105 is homologous to XAC3365 and interacts with D-amino oxidase producing ammonia and hydrogen peroxide as byproducts, as well as acetyl-CoA hydrolase, histidine kinase, TBDR, nuclease, beta-galactosidase, and beta-xylosidase. AKJ12_21575 is homologous to XAC3966, a membrane lipoprotein [16]. According to STRING, this protein interacts with T2SS protein GspL, cellulase, glycosyltransferase, aklaviketone reductase, and 2-keto-4-pentenoate hydratase. AKJ12_07070 is a secreted protein homologous to XAC0825. This protein shows co-occurrence with a peptidase, peptidoglycan-binding protein, endonuclease, ribonuclease, hemin and sugar transport proteins. AKJ12_16480 is a secreted protein homologous to XAC3844 located in the same genomic region as *lysM*, a peptidoglycan-binding protein, and *queF*, a NADPH-dependent reductase.

### 2.3. Functional Enrichment Analysis

We performed an enrichment analysis by gene ontology (GO) to further identify relevant biological processes associated with chorismate mutase in the Xaj417 strain and the plant response to the mutant in walnut fruit. The analysis of the 534 increased proteins in XajCM revealed processes significantly associated (FDR ≤ 0.05) with ribosomal small unit assembly, regulation of cellular component organization, and organonitrogen compound catabolism (Table S4). Table 2 shows the main biological processes based on fold-enrichment (FE). In this analysis, besides ribosomal small subunit assembly, leucine biosynthetic process and gluconeogenesis were also highly enriched in the mutant (FE > 6). Other relevant biological processes increased in the mutant were arginine metabolic processes, response to oxidative stress, tricarboxylic acid cycle (TCA), and siderophore transmembrane transport. These results were also reflected in the GO-term analysis of the molecular functions, complete cellular components, PANTHER pathways, and protein classes. The most significant GO-terms for molecular function in the mutant compared with XajWT were oxidoreductase activity, acting on a sulfur group of donors, NAD, and ATP binding functions. rRNA binding and structural constituent of ribosome was also a function with high fold-enrichment. The cellular component analysis showed GO-terms associated with TCA enzyme complex. GO-terms associated with PANTHER pathways showed leucine biosynthesis as significant and with the highest FE. There were eight significant protein classes associated with transcription, translation and oxidation–reduction reactions.

A similar functional enrichment analysis was performed in the proteome comparison of walnut (Jr) fruit inoculated with either CM mutant or the Xaj417 WT, but only the set of proteins that were differentially abundant that either increased or decreased were analyzed. The polygalacturonase inhibitor 1 protein (PGIP) was the most abundant protein in the mutant. A total of five proteins were significantly decreased in the walnut tissues inoculated with CM mutant (Table 3). The most decreased protein in response to CM compared to WT was a plant natriuretic peptide, an EG45-like domain containing protein 2. This protein was associated with the GO-term biological process systemic acquired resistance (SAR). Molecular functions included GO-terms associated with metal ion binding, including zinc and copper. Conversely, there were ten proteins that were significantly increased in Jr inoculated with the mutant. GO-terms for biological processes showed that half of them were associated with defense response, two with response to abscisic acid, two carbohydrate metabolic processes, and one associated with hydrolase activity and osmotic stress. Molecular functions also included metal binding and polygalacturonase inhibitor activity. In the categories of GO-terms for cellular components, most of the proteins, that both decreased or increased, were associated with apoplast or extracellular compartments.

## 3. Discussion

WB affects walnut productivity worldwide due to the endemic presence of Xaj in orchards [17]. The intense use of copper-based pesticides to prevent disease incidence has led to development of copper-resistant strains such as Xaj417 isolated in California [9]. The availability of the complete genome sequence of both host and pathogen in this pathosystem has enabled a deep profiling of the molecular players involved in disease development. A comparative proteomic analysis of symptomatic vs. healthy walnut fruit tissues identified 67 type II effectors including chorismate mutase confirming its expression during infection in planta [12]. Chorismate mutase is a key enzyme in the shikimate pathway responsible for aromatic amino acid synthesis [18]. This enzyme was also identified as one of the seven unique conserved secreted virulence factors involved in immune dysfunction in plant pathogenic Xanthomonadaceae [4]. Although the role of the secreted chorismate mutase in virulence is unknown, Xoo knockout mutants were shown to be hypervirulent in rice [6]. In this work, we describe the use of a secreted CM knockout mutant of Xaj417 (AKJ12_RS15475 – XajCM) to define its role in blight disease development.

Proteomic analysis by LC/MS-MS of walnut hulls inoculated with Xaj WT or XajCM revealed that all differentially abundant proteins found in mutant-inoculated samples were increased. The mutation in XajCM resulted in a hypervirulent phenotype and colonization advantage in planta suggesting that CM could be an evolutive gain to propagate the pathogen and regulate virulence. According to the Pathogen–Host Interaction database (PHI-database), there are 29 *Xanthomonas* genes described so far that result in a hypervirulent phenotype when knocked out (Table S5). Disruption of virulence suppressor genes increases pathogen virulence [19]. The presence of a gene that negatively regulates virulence might favor host survival and thus transmission to susceptible hosts offering an evolutive adaptation. In a broader sense, the conservation of a system that negatively regulates virulence implies a counter adaptive consequence for loss-of-function mutations.

**Figure 4:**
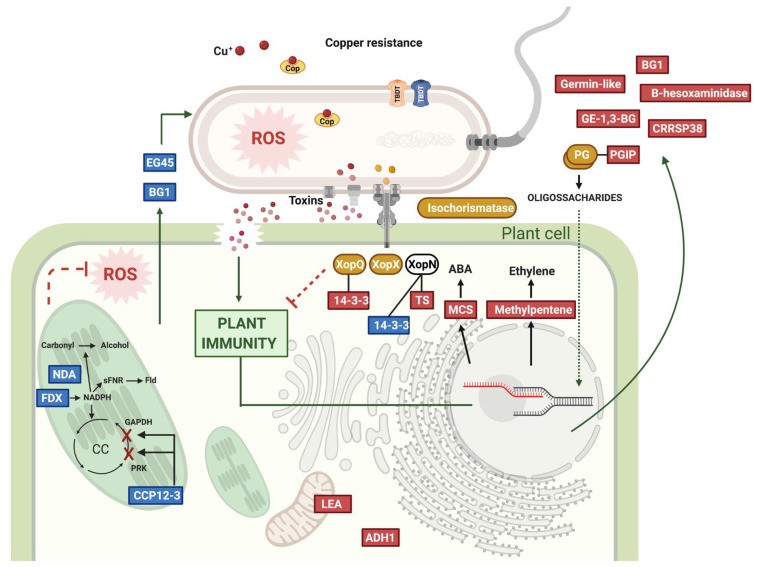
Model of *Xanthomonas arboricola* pv. *juglandis* (Xaj) and *Juglans regia* (Jr) interactome. Xaj (oval shape) and Jr (rectangular shape) proteins. Proteins with increased abundance are shown in red and yellow to represent proteins from Jr and Xaj respectively. Jr decreased proteins are represented in blue rectangles. EG45: EG45-like domain containing protein, BG1: probable glucan endo-1,3-beta-glucosidase, NDA: NADPH-dependent alkenal/one oxidoreductase (AtAOR), FDX: ferredoxin-2, CCP12-3: Calvin cycle protein CP12-3, TBDT: TonB-dependent transporter, PG: polygalacturonase, PGIP: polygalacturonase-inhibitor, GE-1,3-BG: probable glucan endo-1,3-beta-glucosidase At4g16260, CRRSP38: cysteine-rich repeat secretory protein 38, 14-3-3: 14-3-3-like protein, TS: thiamine synthetase, MCS: molybdenum cofactor sulfurase, LEA: late embryogenesis abundant proteins. ADH1: alcohol dehydrogenase class-P. This figure was created in BioRender.com (accessed on 1 September 2021).

Curiously, most of the *J. regia* protein levels were not affected by inoculation with XajCM compared to Xaj WT, suggesting that the host responds similarly to both (Figure 3A). Among the 3296 Jr proteins detected, only ten are significantly increased in XajCM inoculations, including PGIP as the most abundant and two germin-like proteins, while five are decreased compared with XajWT inoculations (Table 3). Germin-like proteins play an important role in the regulation of defense in *Gossypium hirsutum* plants [20]. The overexpression of these proteins in *Arabidopsis* or their silencing in cotton resulted in the activation or suppression of jasmonic acid-mediated signaling, respectively. Similarly, in our study, all 15 Jr differentially abundant proteins in the XajCM mutant inoculations are associated with stress and defense responses (Table 3). Two of the five down-regulated proteins are secreted and associated with SAR and defense responses (EG45 and BG1), while the other three are located in the chloroplast regulating NADPH production leading to a decrease in ROS response (Figure 4). In *Arabidopsis*, the EG45-like domain-containing protein AT4G30380 encodes a PNP-A (Plant Natriuretic Peptide A), a class of systemically mobile molecules distantly related to expansins. PNP-A is secreted into the extracellular space and co-expression analyses using microarray data suggest that PNP-A may function as a component of plant defense response, SAR in particular, and could be classified as a newly identified PR protein [21]. EG45-like protein may also interact with glucan-1,3-beta-glucosidase according to STRING. In contrast, the increased abundance of PGIP demonstrates a plant defense mechanism against cell wall degradation that favors bacterial colonization. Constitutive expression of PGIP confers resistance against phytopathogens and may also aid in defense against herbivorous beetles [22,23,24]. These studies support our proteomic analyses that show PGIP as the most abundant protein during XajCM infection of walnut hull tissues. Other proteins that increased during infection were cysteine-rich repeat secretory protein 38, late embryogenesis abundant protein (LEA), probable glucan endo-1,3-beta-glucosidase, 1,2-dihydroxy-3-keto-5-methylthiopentene dioxygenase 3, alcohol dehydrogenase class-P (ADH), beta hexosaminidase 1, and molybdenum cofactor sulfurase (Figure 4). In pea, LEA functions as a mitochondrial membrane protectant against protein aggregation due to desiccation or osmotic stresses associated with low temperature. LEA proteins are particularly protective of mitochondrial membranes against dehydration damage [25]. ADH is involved in cold stress regulation in sugarcane and it plays a critical role in hypoxic stress tolerance [26,27].

Among the differentially abundant proteins in the host plant, we highlighted the 14-3-3 protein family composed of highly conserved dimeric proteins that recognize a well-defined phosphorylated motif regulating several cellular processes, ranging from metabolism to transport, growth, development, and stress response [28]. 14-3-3 proteins and the FHA domain-containing proteins are the only phospho-binding regulators identified so far in plants [29]. Many studies show differential regulation of 14-3-3 proteins in response to pathogen recognition and their interaction with known regulators of plant immunity and as targets of pathogen effectors [30,31,32]. The Jr genome codes for 20 proteins from the 14-3-3 protein family. Among Jr proteins identified in the proteome, 13 belong to this protein family, three being increased and ten being decreased in the interaction with XajCM mutant. Two tomato 14-3-3 proteins (TFT1 and TFT4) were shown to be targeted by the effectors XopN and XopQ, respectively, promoting virulence by suppressing PTI and ETI respectively [33,34,35]. Interestingly, their homologs in Jr (Jr07_04760_p1 and Jr10_04970_p1) have, respectively, decreased and increased abundance in the mutant while the effector XopN is not differentially abundant and XopQ is increased in XajCM (Figure 4). PTI activation constitutes the first layer of plant defense to avoid pathogen colonization, resisting a broad range of pathogens by recognition of conserved pathogen structures, while ETI is a more robust and faster pathogen-specific defense response. Our results show the importance of the increased expression of XopQ targeting the Jr TFT4 homolog (Jr10_04970_p1) to promote virulence by suppressing ETI. In rice, XopQ interacts in yeast and in planta with two rice 14-3-3 proteins, Gf14f and Gf14g, and the 14-3-3 protein GF14e negatively affects cell death and disease resistance [31,32,33,34]. Its homolog in Jr (Jr12_02590_p1) is increased upon interaction with the mutant compared to the WT bacteria, possibly leading to increased disease susceptibility. As a result, this gene could be a promising target for gene silencing approaches for disease resistance in walnuts. GF14e-silenced rice plants showed high levels of resistance to a virulent strain of Xoo and a necrotrophic fungal pathogen *Rhizoctonia solani* [31].

From the pathogen’s perspective, a total of 676 proteins, approximately 16.2% of the Xaj genome, were identified in the infected walnut proteome. All differentially abundant proteins in the Xaj CM mutant were increased. Polygalacturonase, one of the first enzymes to be secreted by pathogens during infection, was the most abundant protein in XajCM. Polygalacturonan is a component of the pectin fiber network that comprises plant cell walls. By secreting endo-polygalacturonase, Xaj can degrade the cell wall fibers allowing access to internal plant tissues and perhaps internalization into the bacterial cell through TBDR for their use as a carbon source. Among the 236 proteins that increased in the mutant with FDR ≤ 0.05 (Table S3) it is important to highlight 14 hypothetical proteins (Table 1), 17 TBDR, isochorismatase, and 21 degrading enzymes including peptidases, polygalacturonases, hydrolase, endoglucanase, serine protease, enolase, esterase and cellulase. Serine protease facilitates penetration and efficient dissemination [36]. Under stress, the production of SA from isochorismate is favored in plants [3]. In contrast, Xaj secretes isochorismatase as a counter defense mechanism. Iron uptake receptors, transporters and siderophores such as ferritin and enterobactin are among the 60 most abundant proteins (AKJ12_RS14135/RS07080/RS15375/RS13500/RS16595).

Intriguingly, ferredoxin is a siderophore produced to sequester iron in competition with strategies evolved in the host organism, such as nutritional immunity, as well as to compete with other microorganism members of the microbiome associated with the plant host [37]. The Tol-Pal system proteins YbgF, TolB and TolC were also increased in the XajCM mutant. In Gram-negative bacteria, this energized system has interlinked roles in cell division by coordinating the restructuring of peptidoglycan at division sites and stabilizing the connection between the outer membrane and underlying cell wall [38]. In *Xylella*, Tol-Pal genes are overexpressed during biofilm formation [39]. Finally, XopQ and XopX are increased in the XajCM mutant, and XopN is not differentially abundant (FDR = 0.66). In rice, XopQ-XopX interact in planta and co-expression induces rice immune responses by interaction with 14-3-3 proteins GF14d and GF14e [40]. The homologs in Jr are Jr01_07220_p1 (+1) and Jr10_04970_p1 and are differentially abundant in interaction with the mutant as well (Figure 4).

The secretome analysis of *Xanthomonas citri* revealed chorismate mutase as a potential virulence factor [41]. Our results demonstrated that although the plant response is similar, with only 15 differentially abundant proteins compared to inoculations with the wild type phytopathogen, XajCM presents increased abundance of most of the proteins identified. GO analysis of the 534 increased proteins in XajCM infected tissues revealed processes significantly associated (FDR ≤ 0.05) with breakdown of organonitrogen compounds and regulation of cellular component organization which may alter membrane integrity resulting in abnormal cell membranes which could explain changes in XajCM cellular organization observed in the cell culture microscopy (Figure 1B). Enrichment in biological processes such as leucine biosynthetic process, gluconeogenesis, arginine metabolic processes, response to oxidative stress, TCA, and siderophore transmembrane transport corroborates the results obtained by proteomics and phenotyping. In Xoo, biosynthesis of amino acids such as leucine and arginine are essential to its pathogenicity. In addition, leucine could stimulate virulence-related responses and regulate Xoo pathogenicity [42]. GO-terms associated with PANTHER pathways showed leucine biosynthesis with the highest FE.

Finally, the 14 hypothetical proteins increased (FDR ≤ 0.05) in tissues infected with the mutant (Table 1) are homologs of proteins associated with virulence and adaptation. AKJ12_18130 is homologous to XAC0292. This protein is in the same genomic region as *hprB* which plays an important role in motility and biofilm formation in *Xanthomonas* [43]. AKJ12_01945 is a secreted protein homologous to XAC2370, an endopeptidase that contains a plant inducible promoter (PIP)-box suggesting its role as a *hrp* regulon candidate. In addition, mutation in this gene induces hypersensitive response (HR) in grapefruit and tomato [44]. AKJ12_04530 is homologous to an outer membrane protein required for virulence during infection and is involved in copper homeostasis and *hrp* gene expression in *Xanthomonas* [45]. AKJ12_19795 is a secreted protein annotated as Omp1 in *X. citri*, and its absence reduces chemotaxis, biofilm and virulence [46]. AKJ12_01700 is a secreted protein homologous to IA64_08630 from *X. arboricola* pv. *celebensis*, which may interact with glutamine cyclotransferase, a protein known to be co-expressed and interact with proteins belonging to the DEAD box helicase family such as RhlB and DeaD involved in ribosome biogenesis, mRNA degradation and translation initiation [47]. AKJ12_15755 is located close to a gene that codes for a Na+/H+ antiporter known to interact with glutathione-regulated potassium-efflux system protein KefB, and a multidrug ABC transporter ATP-binding protein [48]. AKJ12_09885 is a putative secreted lipoprotein homologous to XAC1761 and interacts with proteins from the outer membrane protein assembly complex BamABCDE, which is involved in the assembly and insertion of beta-barrel proteins into the outer membrane [49]. This protein also was characterized as one of the most abundant proteins in outer membrane vesicles of *X. citri* [50] and it is secreted only by the *hrpB4* mutant grown in XAM1 medium [41]. AKJ12_17400 is homologous to XAC0419, annotated as HPF/RaiA family ribosome-associated protein in *X. arboricola*, a stress-response protein that binds the ribosomal subunit interface and arrests translation by interfering with aminoacyl-tRNA binding to the ribosomal A site [51]. This ribosome-associated inhibitor protein stabilizes bacterial ribosomes under stress, stationary phase, and normal growth conditions and may be correlated with T3SS [51]. AKJ12_14465 is a secreted protein homologous to XAC3439, a protein regulated by diffusible signal factor (DSF)/rpf genes [52]. The gene coding this protein is in the same genomic region as *hppA*, a proton pump that utilizes the energy of pyrophosphate hydrolysis as the driving force for proton movement across the membrane to generate a proton motive force. AKJ12_07680 is homologous to the lipoprotein ElpS, a protein involved in the mobilization of inorganic phosphate [53]. This protein interacts with T2SS, three TBDR, and GuaA and GuaB from the guaAB operon involved in purine salvage pathways for synthesis of DNA and RNA from the host environment [54].

Taken together, our proteomic results corroborate chorismate mutase as a possible *Xanthomonas* virulence suppressor protein. XajCM adapts to the host microenvironment, sequestering iron, responding to redox conditions, increasing energy production and intermediary metabolism. In addition, understanding the molecular mechanisms and biological markers for disease resistance and susceptibility in both host and pathogen could lead to promising approaches for gene silencing or overexpression for disease resistance in walnuts, as exemplified for Jr GF14e homolog and PGIP, respectively. The results presented contribute to a deeper understanding of the role of secreted proteins and their contribution to underlying mechanisms leading to walnut blight disease that could lead to the identification of novel targets for therapy, including genome editing that could lead to the development of broad-spectrum resistance.

## 4. Materials and Methods

### 4.1. Generation of the XajCM Mutant

To disrupt monofunctional chorismate mutase (AKJ12_RS15475), a mutagenesis cassette was synthesized (GenScript, NJ USA) by inserting a kanamycin resistance gene (Tn903 aminoglycoside transferase) within XajCM’s lysine residue at the coding region amino acid position 163, a predicted residue for the catalytic triad. The flanking homology region of this cassette consisted of the entire open reading frame of AKJ12_RS15475 (163bp at 5′ and 407 bp at 3′). The synthesized mutagenesis cassette (pUC57-XajCM_KanR) was electroporated into *Xanthomonas arboricola* pv. *Juglandis* 417 wildtype (Xaj WT) competent cells grown in the NYGA medium as previously described [26]. Transformant double crossover events were confirmed via gel electrophoresis and sequencing using oligonucleotide primers, Fwd: ATGACCCGCACGATCGATG and Rev: TCAGTGGCAGAAATCGCCCA, designed to anneal to the 5′ and 3′ regions of the AKJ12_RS15475 (Figure S1). The verification of PCR amplicons was established by applying 8 μL of the PCR product in 0.8% (*w*/*v*) agarose gel.

### 4.2. Scanning Electron Microscopy

Morphological characterization was performed by using the Quattro environmental electron microscope (Thermo Fisher Scientific, Bedford, MA, US) at the UC Davis Tupper Hall Electron Microscopy Lab Facility and UC Davis Kemper Hall Advanced Materials Characterization and Testing laboratory (AMCaT) operating at 20 kV. Cells were fixed overnight in 0.1 M sodium phosphate buffer with 2.5% glutaraldehyde and 2% paraformaldehyde. Subsequently, cells were centrifuged and then resuspended and rinsed in 0.1 M NaH_2_PO_4_. Then, a 12 mm glass coverslip was prepared by adding a few drops of aqueous 0.1% polylysine solution. After one hour, the polylysine was removed and the sample was added to the coverslip and let sit for one hour. Afterward, the solution was removed, the coverslip was rinsed in 0.1 M NaH2PO4, and dehydrated in increasing concentrations of EtOH for 10 min each (30% EtOH, 50% EtOH, 70% EtOH, 95% EtOH, 100% EtOH). Dehydration with 95 and 100% EtOH was repeated three times. Samples were dried using Tousimis 931 GL Super Critical Autosamdri, mounted onto aluminum stubs, and coated with gold using Pelco Auto Sputter Coater SC-7.

### 4.3. Growth Curve, Bacterial Inoculation and Plant Material

Xaj WT and XajCM were grown in YEP, transferred to liquid media, adjusted to an OD of 0.1 and measured at 600 nm in a total volume of 10 mL. Cultures were incubated in a 28 °C shaker at 220 rpm. Growth was monitored at regular intervals by OD_600_ measurements and by plating out for viable counts for 18 h. For inoculation of walnut fruit, Xaj WT and XajCM were grown in YEP plates for two days and cultured in 20 mL of YEP media overnight by shaking at 200 rpm [55]. Kanamycin at 50 μg/mL was used for selection of the mutant in plates and for liquid cultures. Eight nuts per treatment were collected from a Chandler tree at the Hutchison field (Davis, CA, USA) and washed with distilled (DI) water before inoculation. Cultures were centrifuged for 10 min at 4000× *g*, the supernatant was discarded, and the pellet washed with 15 mL of 5 mM MgCl_2_ with 0.1% Break-Thru S 240 (Evonik Industries, Essen, Germany). The OD_600_ was measured, the cultures were centrifuged under the same conditions and the pellet was washed with 10 mL of 5 mM MgCl_2_ with 0.1% Break-Thru. After the second wash, the volumes from the two replicates were mixed. OD_600_ was measured and then adjusted to 1 × 10^8^ bacterial cells/mL in 245 mL of 5 mM MgCl_2_ with 0.1% Break-Thru. For inoculation, walnut fruits were submitted to vacuum in a 1 × 10^8^ bacterial solution for 1 min (30 s vacuum on and 30 s under pressure) and placed to air dry on tissue paper before they were placed in two humidity boxes containing eight nuts per treatment each, under controlled light as previously described [12]. The development of symptoms was monitored for ten days and then samples were collected for proteomics and microscopy. The samples that were used for proteomics and for microscopic analysis were harvested on day 7- and day 10-post-inoculation (dpi), respectively. For proteomics, the samples were divided into three pools (technical replicates) containing epidermal peels of hull tissues obtained from two walnut fruit, one from each humidity box.

### 4.4. Sample Preparation for Proteomic Analysis

Peeled hull tissues from inoculated nuts were pooled. Every two walnut nuts provided material for one biological replicate with a total of three biological replicates per inoculation (Mock, Xaj WT, and XajCM). The proteomic analysis of these tissues was performed in an identical manner to that described (Mock and Xaj WT) previously [12]. Protein extraction, quantification, digestion (trypsin), acidification, clean up, and labeling were performed at the UC Davis Proteomics Core Facility and fractionated using the Pierce™ High pH Reversed-Phase Peptide Fractionation Kit (Thermo Fisher Scientific) for LC-MS/MS analysis. Tandem mass tag (TMT) system TMT10plex™ Label Reagent Set (Thermo Fisher Scientific) was used for pooling samples together according to the manufacturer protocol.

### 4.5. Xaj417 Cell Count by Digital PCR (dPCR)

All assays involving cell count of Xaj 417 by dPCR were carried out following the protocols described by Sagawa, 2021 [56]. DNA was extracted with the DNeasy Plant Mini™ Kit (Qiagen, Germantown, MD, US). Reactions were loaded into a QIAcuity 8.5 K 96-well Nanoplate (250021) and loaded onto a QIAcuity 4-plate 5-plex instrument. The DNA marker and probes used were XAJ1 (KU577316.1) as previously described [57] and the reporter TET fluorescein dye at the 5′ end and the quencher Iowa Black FQ at the 3′ end. The internal ZEN quencher was used in addition to the 3′ quencher Iowa Black FQ.

### 4.6. Data Analysis and Raw Data Processing

Proteomic data files were analyzed using MS Convert from the Proteowizard toolkit [58] and Python scripts from the PAW (proteomic analysis workflow) pipeline [59] as previously described [12]. The protein database combined proteins from *Xanthomonas arboricola* pv. *juglandis* 417 (4178 sequences downloaded from NCBI RefSeq NZ_CP012251.1) and *Juglans regia* (41,103 sequences downloaded from NCBI assembly GCF_001411555.2).

### 4.7. Quantification and Statistical Analysis

The scored abundance data was uploaded to MetaboAnalyst 4.0 (http://www.metaboanalyst.ca (accessed on 1 September 2021)) for principal component analysis (PCA) and partial least squares-discriminant analysis (PLS-DA) to identify proteins contributing to the discrimination between different treatments, as shown in Figures S3–S6. The variables were mean-centered and divided by the standard deviation of each for data scaling, and the Leave One Out Cross-Validation (LOOCV) method was performed to evaluate the quality of the resulting statistical models by considering the diagnostic measures R2 and Q2 [60]. PLS-DA measures the Variable Importance in Projection (VIP) score to estimate the importance of each variable in the projection used in a PLS model and is often used for variable selection. All assays involving quantification and statistical analysis were performed following the protocols described by Sagawa et al., 2021 [56].

### 4.8. Functional Enrichment, Protein Subcellular Localization, and Metabolic Pathways Analysis

The PANTHER classification system was used to perform protein functional and gene ontology (GO)-term enrichment analysis [61]. *Xanthomonas campestris* pv. *campestris* strain ATCC 33913 (taxid:190485) orthologs for Xaj proteins and *Vitis vinifera* orthologs for Jr proteins were used in the PANTHER Overrepresentation Test (Released 20200728). Differentially abundant proteins were determined based on fold change (FC) ≥ 2 and FDR ≤ 0.05. Subcellular localization of Xaj and Jr proteins were predicted by BUSCA [62], SignalP [63] and Phobius [64]. KEGG pathways [65] were used to identify the metabolic pathways associated with significant proteins. Protein–protein interaction prediction was done using STRING [66] and reannotation of hypothetical proteins using BLAST [67].

## Figures and Tables

**Table 1:** Differentially abundant hypothetical proteins in XajCM compared to Xaj417.

Protein Name/Locus Xaj417	VIP Score	Homology	SignalP	BUSCA	log2 FC (CM/WT)	Direction	*p*-Value	FDR	Candidate
hypothetical protein AKJ12_14105	6	XAC3365	OTHER	Cytoplasm	1.48	up	0.000000	0.000071	high
hypothetical protein AKJ12_21575	17	XAC3966	OTHER	Cytoplasm	1.22	up	0.000018	0.001228	high
hypothetical protein AKJ12_07070	23	XAC0825	SP (Sec/SPI)	SP-Extracellular space	1.11	up	0.000001	0.000126	high
hypothetical protein AKJ12_18130	32	XAC0292	OTHER	Cytoplasm	1.06	up	0.000000	0.000021	high
hypothetical protein AKJ12_01945	33	Endopeptidase (XAC2370)	SP (Sec/SPI)	SP-OM-Beta Strand	1.15	up	0.000000	0.000007	high
hypothetical protein AKJ12_04530	40	Omp (XAC1347)	OTHER	Cytoplasm	1.07	up	0.000025	0.001457	high
hypothetical protein AKJ12_19795	71	Omp1	LIPO (Sec/SPII)	SP-Extracellular space	0.96	up	0.000264	0.007913	high
hypothetical protein AKJ12_01700	74	IA64_08630	SP (Sec/SPI)	SP-Extracellular space	0.90	up	0.000000	0.000010	high
hypothetical protein AKJ12_15755	97	PXO_03051	OTHER	PM-Alpha Helix	0.83	up	0.002633	0.043419	med
hypothetical protein AKJ12_16480	184	XAC3844	OTHER	PM-Alpha Helix	0.73	up	0.000347	0.009915	high
hypothetical protein AKJ12_09885	237	XAC1761	LIPO (Sec/SPII)	SP-OM-Beta Strand	0.67	up	0.000476	0.012267	med
hypothetical protein AKJ12_17400	280	RaiA (XAC0419)	OTHER	Cytoplasm	0.70	up	0.001134	0.024155	med
hypothetical protein AKJ12_14465	437	XAC3439	SP (Sec/SPI)	SP-Extracellular space	0.52	up	0.001445	0.028278	med
hypothetical protein AKJ12_07680	474	ElpS (XAC0692)	OTHER	Cytoplasm	0.51	up	0.002401	0.040406	med

**Table 2:** GO biological process and molecular function for increased Xaj protein abundance in Xaj CM/WT (FDR ≤ 0.05). FE: fold enrichment. Complete table with GO-terms is available in the supplementary material (Table S4).

GO Biological Process	Xcc (4126)	Input (534)	Input (Expected)	Input (Over/Under)	FE	Raw *p*-Value	FDR
regulation of cellular component organization (GO:0051128)	8	6	1.04	+	5.79	2.94 × 10^−3^	4.64 × 10^−2^
organonitrogen compound catabolic process (GO:1901565)	91	24	11.78	+	2.04	2.79 × 10^−3^	4.46 × 10^−2^
DNA metabolic process (GO:0006259)	188	10	24.33	-	0.41	2.77 × 10^−3^	4.46 × 10^−2^
purine nucleoside bisphosphate metabolic process (GO:0034032)	22	10	2.85	+	3.51	2.22 × 10^−3^	3.74 × 10^−2^
gluconeogenesis (GO:0006094)	7	6	0.91	+	6.62	1.86 × 10^−3^	3.24 × 10^−2^
response to oxidative stress (GO:0006979)	31	13	4.01	+	3.24	9.10 × 10^−4^	1.85 × 10^−2^
glycolytic process (GO:0006096)	16	10	2.07	+	4.83	3.45 × 10^−4^	7.82 × 10^−3^
leucine biosynthetic process (GO:0009098)	5	5	0.65	+	7.73	2.99 × 10^−3^	4.67 × 10^−2^
tricarboxylic acid cycle (GO:0006099)	20	14	2.59	+	5.41	8.93 × 10^−6^	3.08 × 10^−4^
siderophore transmembrane transport (GO:0044718)	35	19	4.53	+	4.19	3.75 × 10^−6^	1.38 × 10^−4^
**GO molecular function**	**Xcc (4126)**	**Input (534)**	**Input (expected)**	**Input (over/under)**	**FE**	**Raw *p*-value**	**FDR**
oxidoreductase activity, acting on a sulfur group of donors (GO:0016668)	12	8	1.55	+	5.15	1.01 × 10^−3^	4.56 × 10^−2^
oxidoreductase activity, acting on the CH-OH group of donors (GO:0016616)	47	21	6.08	+	3.45	1.23 × 10^−5^	8.32 × 10^−4^
siderophore uptake transmembrane transporter activity (GO:0015344)	34	19	4.4	+	4.32	2.71 × 10^−6^	2.10 × 10^−4^

**Table 3:** Differentially abundant proteins in Jr with FDR ≤ 0.05.

Protein Name	Log2 FC (CM/WT)	Direction	*p*-Value	FDR	Candidate	GO: Biological Process	GO: Cellular Component	GO: Molecular Function	KW: Ligand
PGIP	2.520	up	0.000000	0.000000	high	Defense response	Extracellular region (EC)	Polygalacturonase inhibitor activity	na
Cysteine-rich repeat secretory protein 38	0.770	up	0.000000	0.000002	high	Response to abscisic acid	Extracellular region (EC)	na	na
Germin-like protein subfamily 1 member 7	1.446	up	0.000001	0.000114	high	Defense response	Apoplast; cell wall	Mg ion binding; nutrient reservoir activity	Mg-Metal-binding
Germin-like protein subfamily 1 member 13	0.673	up	0.000218	0.006936	high	Defense response	Apoplast; cell wall	Mg ion binding; nutrient reservoir activity	Mg-Metal-binding
Late embryogenesis abundant protein At1g64065	0.506	up	0.000330	0.009552	high	Abiotic stress	Integral component of membrane	na	na
Probable glucan endo-1,3-beta-glucosidase At4g16260	0.477	up	0.000360	0.010207	med	Carbohydrate metabolic process; defense response	Anchored component of PM; EC	Hydrolase activity; polysaccharide binding	na
1,2-dihydroxy-3-keto-5-methylthiopentene dioxygenase 3	1.672	up	0.001201	0.024840	med	Methionine metabolic process	Cytoplasm; nucleus	Iron ion binding	Fe-Metal-binding
Alcohol dehydrogenase class-P (AtADH)	0.585	up	0.001429	0.028188	med	Response to hypoxia, abscisic acid, estradiol, hydrogen peroxide, and osmotic stress	Cytosol; plasma membrane	NAD activity; nucleotide and zinc ion binding	Metal-binding
Beta-hexosaminidase 1	0.327	up	0.002061	0.036378	med	Carbohydrate metabolic process	Cytosol; vacuole	Beta-N-acetylhexosaminidase activity	na
Molybdenum cofactor sulfurase (MCS; MOS; MoCo sulfurase)	0.456	up	0.002370	0.040231	med	ABA biosynthesis; auxin-signaling pathway; defense response; stomatal movement	Intracellular	Molybdenum ion binding; selenocysteine lyase	Pyridoxal phosphate
EG45-like domain containing protein 2	−1.155	down	0.000041	0.002101	high	SAR	Apoplast; cell wall	na	na
Ferredoxin-2, chloroplastic (AtFd2)	−0.633	down	0.000389	0.010674	med	Photosynthetic electron transport chain	Chloroplast	Metal ion binding	Metal-binding
NADPH-dependent alkenal/one oxidoreductase, chloroplastic (AtAOR)	−0.389	down	0.001066	0.023011	med	Oxidation-reduction process	Apoplast; chloroplast; thylakoid	Enone reductase activity; zinc ion binding	NAD
Probable glucan endo-1,3-beta-glucosidase BG1	−0.674	down	0.002270	0.038983	med	Carbohydrate metabolic process; defense response	Anchored component of PM; EC	Hydrolase activity; polysaccharide binding	na

## Data Availability

The data that support the findings of this study are openly available, all normalized data used for interpretation of results is available as supplementary material and raw data is available upon reasonable request to authors.

## Supplementary Materials

The following are available online at https://www.mdpi.com/article/10.3390/ijms221910374/s1.
